# Breast Cancer Aptamers: Current Sensing Targets, Available Aptamers, and Their Evaluation for Clinical Use in Diagnostics

**DOI:** 10.3390/cancers13163984

**Published:** 2021-08-06

**Authors:** Kathleen Varty, Connor O’Brien, Anna Ignaszak

**Affiliations:** Department of Chemistry, University of New Brunswick, Fredericton, NB E3B 5A3, Canada; kvarty@unb.ca

**Keywords:** cancer, cancer detection, aptamers, diagnostics, breast cancer, cancer biomarkers, sensors, aptasensors, electrochemical sensing, biosensors

## Abstract

**Simple Summary:**

Aptamers, which are short sequences of oligonucleotide or peptides, have become an increasingly popular tool within the field of oncology. This increase in usage is attributed to an aptamer-specific design that allows for high degrees of target specificity and high binding affinity, having thus become of specific interest for the design of sensing platforms. Herein, we look to present the reader with all of the prominent known aptamers and their receptors for the detection of breast cancer. Our hope is that with the knowledge presented in this review, researchers will have a hub to explore the advantages and disadvantages of each aptamer and their target, thus allowing for informed project design and a foundation to build upon.

**Abstract:**

Breast cancer is the most commonly occurring cancer in women worldwide, and the rate of diagnosis continues to increase. Early detection and targeted treatment towards histological type is crucial to improving outcomes, but current screening methods leave some patients at risk of late diagnosis. The risk of late diagnosis and progressed disease is of particular concern for young women as current screening methods are not recommended early in life. Aptamers are oligonucleotides that can bind with high specificity to target molecules such as proteins, peptides, and other small molecules. They are relatively cheap to produce and are invariable from batch to batch, making them ideal for use in large-scale clinical or screening programs. The use of aptamers for breast cancer screening, diagnosis, and therapeutics is promising, but comparison of these aptamers and their corresponding biomarkers for use in breast cancer is significantly lacking. Here, we compare the currently available aptamers for breast cancer biomarkers and their respective biomarkers, as well as highlight the electrochemical sensors that are in development.

## 1. Introduction

Accounting for 25% of all cancers in women, breast cancer (BC) is the most commonly occurring cancer in women worldwide [1]. BC is a notoriously heterogeneous disease, with the World Health Organization recognizing at least 17 subtypes [2]. Traditional classification of breast cancer subtypes focuses on two criteria: the tissue affected and level of invasiveness. Carcinomas, accounting for 99% of breast cancers, arise from the epithelial component of the breast. Sarcomas, the rarer of the two, arise from connective tissue such as bones, nerves, and muscles. After establishing whether the cancer is a carcinoma or sarcoma, one can further separate the cancer subtypes into non-invasive, invasive, and metastatic categories [3]. Metastasis occurs when the cancer spreads to tissue or organs beyond the original site; it is the leading cause of death in cancer patients [4]. The current standard of screening for breast cancer is mammography, clinical breast examination, and routine self-breast examination [5]. For women of all ages and at average risk, these screening methods reduce mortality by 20% [6].

Although traditionally considered a disease of industrialized countries, over half of breast cancer-related deaths occur in mid- to low-income countries. BC diagnosis continues to increase worldwide, most likely due to increased westernization and associated risk factors, such as obesity, associated with such a lifestyle [7,8]. Despite the high rates of incidence, BC generally presents with good outcomes and is curable in 70–80% of cases if caught early and no metastasis has occurred [9]. Although the majority of patients are postmenopausal [10], young women generally present with more aggressive disease and poorer outcomes than the older population. Unfortunately, current screening standards are generally deemed inefficient and sometimes even deleterious in young women, meaning the disease can escape early detection. The inability to detect the disease early in young women is likely responsible for the reporting of poorer clinical outcomes [11].

Palpable breast cancer is only present in about 30% of cases [12], making mammography a key player in screening and early detection. Regrettably, overdiagnosis/false positives are a risk associated with mammography that can result in increased patient anxiety and, at times, unnecessary biopsy. Those with dense breast tissue, approximately 43% of women in high-income countries, are at an even greater risk of overdiagnosis and thus subsequent distress and harm caused by unnecessary intervention [13,14]. Additionally, mammography presents a number of confounding variables such as physician interpretation, poor positioning, and tumor sub-types that can result with as many as 30% of breast cancers going undetected [5]. As such, the search for different screening methods with better specificity and lower false-positive rates are of great interest. 

It is well established in the literature that early detection is essential to improved outcomes and reduced mortality. Ensuring early screening is affordable, timely, and effective care is key to successful implementation of these programs [15]. Aptamers are oligonucleotides, either RNA or single-stranded DNA, that bind specifically to target molecules as a result of their unique folding and 3D composition [16]. In diagnosis, aptamers generally bind with high specificity and affinity, resulting in a conformational change that can be measured and quantified via electrochemical parameters such as resistivity, voltammetry, and amperometry [17,18], as well as through optical methods such as fluorescence and colorimetry [19]. Using aptamers to identify biomarkers of breast cancer provides more information about the characteristics of each patient’s cancer, thus allowing clinicians to make better benefit–risk assessments and provide a better level of care.

Alongside their function within the field of diagnostics, aptamers have also found some success as drugs and drug delivery systems [20]. In the field of therapeutics, many aptamers in literature have been found to act as potent antagonists [21]. Aptamers have also been leveraged as delivery systems to transport drugs specifically to the site of action [22]. Despite the function of aptamers in therapeutics, treatment and drug delivery will not be discussed in depth in this review. 

Aptasensors could also provide an alternative screening option for women in the age groups where standard screening is not recommended. Assessing metastasis through aptamer-based sensing is also a platform of great interest, as metastasis is responsible for most breast cancer deaths and early intervention is crucial [3]. Aptamer-based electrochemical sensing is an ideal candidate for breast cancer screening as they are generally highly sensitive, low cost, and portable [23]. Currently no aptasensors are commercially available for oncological use or disease diagnosis, despite the presence of seemingly functional aptasensors existing in literature that use point-of-care friendly technology such as smartphone linking [24]. In regard to drug delivery, the majority of oncological clinical trials focus on aptamer AS1411 and NOX-A12, neither of which have completed clinical trials past phase II [25].

**Figure 1:**
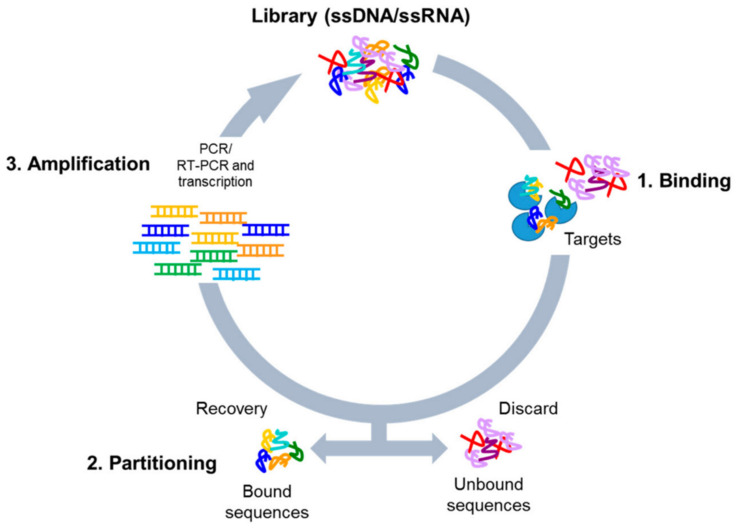
Scheme of the SELEX process [26]. The procedure involves repeated cycles of (1) incubation of the high-complexity library with the targets (binding); (2) removal of unbound sequences and recovery of the bound oligonucleotides (partitioning); (3) amplification of the bound sequences by PCR (for DNA library) or RT-PCR and transcription (for RNA library). (Reproduced with copyright permissions from 10.1002/anie.201001736 accessed on 3 June 2021).

To produce novel aptamers, a method called systematic evolution of ligands by exponential enrichment (SELEX) is used (Figure 1 [26]). To begin, a large library of DNA or RNA is incubated with the target molecule, those that bind to the target are then amplified using PCR. The process is repeated with the PCR product until many aptamers exist, which then can be exploited for their specific properties [27,28]. Compared to other common capture methods such as antibodies, aptamers are more stable, cheaper, faster to produce, and small in size, and there is no batch-to-batch variation [29], again exemplifying their potential for sensing and mass production. 

We acknowledge that a review exists identifying the use of aptamers in breast cancer diagnosis and therapeutics [30]. However, there is a significant gap in the literature comparing aptamers for specific targets against one another. Previous work has focused on modification of the sensing platform, but further exploration of specific aptamer features is needed. Our comparison of aptamers (Table 1) provides another variable to modify in the search for rapid, affordable, and accessible aptamer-based diagnosis and therapeutics for the fight against breast cancer. 

## 2. Breast Cancer Biomarkers and Existing Aptamers

### 2.1. Alpha Estrogen Receptor 

Estrogen receptor α (ERα) and transcriptional activity associated with the receptor is a driving force in approximately 75% of breast cancers [34]. ERα is a ligand inducible transcription factor responsible for estrogen signaling and is primarily found in mammary glands as well as other organs such as ovaries [35,36]. When estrogen binds to ERα, signaling pathways are activated that lead to tumor growth and proliferation in cells that have an excess of these receptors [37]. Targeted hormone therapies for these subtypes of BC exist, but resistance is easily developed [38]; therefore, early identification leads to better overall treatment outcomes.

An aptamer for ERα was only identified as of 2016 by Ahirwar et al. [39]. Appropriately named ERaptD4, this aptamer shows an affinity and specificity similar to that of the currently used ERα antibodies [39]. Current detection of ERα relies on an immunohistochemistry assay that is reliant on the use of antibodies that have been shown to have poorer tissue penetration compared to aptamers, possibly due to their greater molecular weight [40]. ERaptD4 was found to have a relative binding of 78.5% with ERα, nearly double that of the other two top contending aptamers in this study. The aptamer also displayed high selectivity for ERα, regardless of purified receptor or entire cell. Using an aptamer-ELISA against ERα and the ERα ligand binding domain, the group was also able to find that the aptamer binds the ligand domain.

At the time of writing, only one aptasensor has been developed using ERaptD4, by the same group that identified the aptamer. The sensor uses differential pulse voltammetry to achieve a ERα reading with detection limit of 0.001 ng/mL on a gold screen-printed electrode. It only takes 10 min to achieve a reading and due to the nature of the sensor, and the device is easily portable [41]. For these reasons and the importance of hormone resistance in ERα targeted therapies, this is an aptamer worthy of continued research.

### 2.2. EpCAM 

Epithelial cell adhesion molecule (EpCAM) is a transmembrane glycoprotein responsible for cell adhesion, proliferation, and migration. EpCAM can be used as a prognostic factor for multiple cancers; interestingly, high expression for some cancers (such as breast) is associated with poor clinical outcomes, whereas the inverse is true for other cancers (such as thyroid cancer) [42]. EpCAM has been found to be a particularly potent biomarker for breast cancer metastisis, known to be one of the most important factors in patient mortality [43]. CellSearch is the only clinically validated method of detecting EpCAM in metastatic breast cancer patients and works by conjugating EpCAM-specific antibodies with ferrofluid, allowing for magnetic separation. The biggest downfall of this system is that only high EpCAM-expressing cells are detected [44,45]. 

The first aptamer developed to target EpCAM was aptamer EpDT3. A total of three aptamers were developed during the study, but EpDT3 was eventually selected for its high binding affinity to the EpCAM-expressing line Kato III cell line. EpDT3 was able to bind to cells expressing both high and low-medium levels of EpCAM, which is not necessarily beneficial. Binding unselectively means that the aptamer would target both healthy and tumorous cells and could therefore not be used for diagnosis or drug delivery [46].

In 2013 SYL3, a DNA aptamer for EpCAM was developed by Song et al. [47]. The group had previously developed RNA aptamers for EpCAM; however, they were extremely prone to nuclease degradation compared to the DNA aptamer, and as such, little work exists on their use. SYL3 targets the extracellular domain of EpCAM and was seen to bind to EpCAM-positive cell lines but also EpCAM recombinant protein. To determine binding affinity and selectivity, researchers performed tests on breast cancer cell line MDA-MB-231 and blood. The dissociation constant for MDA-MB-231 was 38 ± 9 nM, and SYL3 was found to be selective for EpCAM-positive cells in blood. SYL3 was later optimized in length to produce aptamer SYL3C, nearly half the size of the original aptamer. With reduced length, SYL3C was shown to have better binding affinity then SYL3, with a K_D_ of 38 ± 9 nM for MDA-MB-231 and 67 ± 8 nM for KatoIII cell lines.

The most recent development is aptamer EpApt-siEp, a chimeric short interfering RNA (siRNA). This aptamer was found to show complete tumor regression with no toxicity in animals. EpApt-siEp appears to eliminate EpCAM-positive cancer cells while leaving healthy EpCAM-negative cells. The aptamer also carries a modification (2′F) in the pyrimidines, allowing for nuclease resistance. EpApt-siEP boasts promising therapeutic potential for treatment of breast cancer [48].

The majority of electrochemical sensors in the last five years has focused on detection of EpCAM in serum and blood, rather than the tumor microenvironment [49,50,51]. Recent work has used SYL3C to target circulating tumor cells [52], which express EpCAM on their cell surface [53], with a limit of detection of 10aM being reported by Zhu et al. [50].

### 2.3. Nucleolin

In normal cells nucleolin, a phosphoprotein with RNA recognition motifs, is found primarily in the nucleolus [54]. In cancerous cells, the amount of nucleolin on the cell surface has been found to be abnormally high. This overexpression of nucleolin is associated with poorer patient outcomes as the protein is known to promote carcinogenesis, proliferation, metastasis, and angiogenesis [55]. Nucleolin expression determination with antibodies requires tissue samples, and as such is an invasive procedure [56].

Aptamer AS1411, previously referred to as AGRO100, is the only available aptamer for the detection of nucleolin [57]. AS1411 has been used to detect cell lines MCF-7 [58,59] and MDA-MB-231 [60], but more work should be done to assess the use of the aptamer in real tissue samples. Unlike many other aptamers, AS1411 was not developed using the SELEX process; instead, it was developed with a focus on function over form and based on previous studies that showed the anti-proliferation effects of guanine rich oligionucleotides [61]. AS1411 is known to target nucleolin; however, the specific site of attachment is unknown. It is important to note that we know the aptamer also binds to other proteins such as EEF1A, and as such, is more likely useful for therapeutics other than sensing [62].

In 2019, AS1411 was used to detect CTC in the blood samples of breast cancer patients. Unfortunately, the sensor lacked a large sensing range, indicating that much work is necessary before clinical use [63]. Most interestingly, this aptamer has shown potential for a double sensing platform for ions such as copper [64] and iodine [60]. Whether this duality is beneficial or detrimental is still debatable as it has not yet been used on real clinical samples. We propose that this duality is actually detrimental as it would likely result in a lower specificity due to cross reactivity and produce erroneous readings by detecting both the ion and the biomarker.

## 3. Serum Markers

### 3.1. MUC1

Mucin 1 (MUC1) is a glycoprotein that is both secreted into the extracellular environment and found on the surface of epithelial cells in breast tissue. Secreted MUCs function by forming a mucous membrane to protect organs by forming a physical barrier [65]. Overexpression of this receptor has been found to be correlated with cell proliferation, metastatic potential, and chemoresistance [66]. MUC1 expression on circulating tumor cells (CTC) is indicative of treatment ineffectiveness. Monitoring of MUC1 levels is a useful indicator of breast cancer remission status [67]. Over 90% of breast cancers overexpress MUC1, making it an ideal target for indiscriminate testing, as most patients with cancer will have the biomarker. MUC1 is also overexpressed in other epithelial cancers, and therefore serum samples would only indicate cancer presence but not organ of origin [65].

MUC1 is one of the targets with the most aptamers. Three aptamers exist: aptamer 5TR1 targets the five tandem repeats found within the variable tandem repeat region of MUC1. The developed aptamer was found to have similar binding properties to the respective antibody and actually competes with the antibody for binding [68]. Cross reactivity and specificity with competing analytes in human blood was not assessed and further exploration is needed. 

The same group previously developed an aptamer that targets the cell surface of the MUC1, entitled S2.2. Using competition assays it is believed that the aptamer binds to a similar epitope as the anti-MUC1 antibody. Of all aptamers developed in this study, S2.2 had the lowest K_d_, indicating a higher affinity for the target. The specific segment of attachment for this aptamer is less apparent then the previously mentioned one, and more immunohistochemical studies should be conducted to assess the specificity of the aptamer [69].

The most recent MUC1 DNA aptamer (MA3) was developed by Hu et al. for use in drug delivery to cancerous cells [70]. This aptamer targets an immunodominant peptide epitope. The specificity of the aptamer was tested against albumin, the most abundant protein in blood, and found to be highly specific towards MUC1. Although MA3 has a lower K_d_ then the previously mentioned S2.2, it was found that S2.2 binds with some degree to albumin, meaning it displays a greater cross-reactivity. Although MA3 binds with less affinity, it may be preferable over S2.2, as less cross-reactivity is observed, meaning it is more specific for MUC1.

Using an aptamer based on aptamer 5TR1, a sensor developed by Gupta et al. is able to be reused eight times and is stable for storage for approximately half a month [71]. With a LOD of 1 fg/mL and a run time of 15 min, this sensor would be a great candidate for possible commercialization. Although the sensor was tested on human serum spiked with MUC1, more work needs to be conducted on human serum samples to look at cross-reactivity. This group later expanded on this sensor using bimetallic gold platinum nanoparticles rather than gold exclusively. Although less reusable, this sensor developed by Bharti et al. has a lower LOD at 0.79 fM and can be stored for the same time period [72]. 

### 3.2. Carbohydrate/Cancer Antigen 15-3 (CA 15-3) 

Cancer antigen 15-3 (CA 15-3) is the soluble product of the MUC-1 gene found in varying levels in serum. It is one of the two most common biomarkers used for breast cancer [57]. CA15-3 is currently recommend for use in monitoring metastatic potential in active treatment, assessing prognosis, and early detection of disease progression [73]. Elevated levels of CA15-3 are correlated with poor clinical outcomes and both disease-free and overall survival [73,74]. Development of an aptamer for this target is not critical, as multiple commercial tests are available and in use. Although these testing platforms use antibodies, they both show good analytical performance [75]. In 2017, Wang et al. noted that CA15-3 could be used to distinguish bone metastasis from other types of metastases [76].

Only three aptamers exist for CA 15-3, and they were all developed in 2014 by the same group. The aptamers are entitled Clone 2, Clone 4, and Clone 5. The aptamer with the lowest dissociation constant is Clone 2 with a K_D_ of 45.47 ± 3.415 nM. The aptamer with the next lowest dissociation constant is Clone 4, followed by Clone 5 [77]. The initial research on this aptamer is severely lacking, and no cross-reactivity or specificity studies have been performed outside of those used within sensors. More research should be done using cell lines and ideally, serum focusing on Clone 2, as it has the greatest binding affinity for CA 15-3.

Aptasensors, electrochemical or otherwise, are sparse for CA 15-3, and currently only one electrochemical aptasensor for CA 15-3 has been published. The sensor is dual-platform, sensing both CA 15-3 and CEA using a sandwich-type sensor [78]. Gold nanoparticle three-dimensional graphene hydrogel was used to anchor aptamer CAAp. After CA 15-3 was added, the same aptamer was used as a redox probe to give the sandwich structure described. With an incubation step of an hour, the sensor is not rapid in its current iteration, but a low LOD at 11.2 × 10^−2^ U mL^−1^ and the capacity for dual sensing are promising for future expansion of the work. The lack of aptasensors for this biomarker is likely because it is an antigen, and the inclination is to use an immunosensor. With only one aptamer available for CA 15-3, development of aptasensors for this biomarker is not of particular interest, particularly when antibody-based sensors are better established, and some have shorter processing times [79] and similar LODs to aptasensors [80].

### 3.3. Periostin 

Periostin is a multimodular protein secreted into the tumor microenvironment (TME), known to be overexpressed in BC [81]. Metastasis, chemoresistance, and angiogenesis are some of the cancer-promoting activities that occur with overexpression of periostin [82]. It is important to note that there is a positive correlation between age and periostin expression, and more work needs to be done to obtain a normal range by age [83].

PNDA-3 is the only aptamer available for detection of periostin. To the authors knowledge, no commercially available tests exist for the detection of periostin as a BC biomarker. The Elecsys^®^ Periostin immunoassay is the only test currently under development but is being used to target periostin in asthma rather than BC [84]. This is likely a result of the high variation of periostin levels due to age and other factors [83]. PNDA-3 is a DNA aptamer that binds to the third or fourth FAS1 domain of periostin in its secreted form [85]. PNDA-3 exhibits high affinity for periostin with a K_d_ of 1.07 nM and inhibits tumor growth in vivo but less effectively in vitro. Using a mouse model, researchers showed that PNDA-3 displayed significant inhibition of the metastasis of tumors, implicating great potential for therapeutic use [86]. If a reference range for normal levels of periostin can be established, PNDA-3 could provide a promising sensor for breast cancer as the aptamer has also been found to inhibit breast cancer growth and metastasis [86], lending to the possibility for use of the aptamer in sensing and treatment. 

### 3.4. Carcinoembryonic Antigens (CEA) 

Carcinoembryonic antigens (CEA) are glycoproteins found both in the tumor microenvironment (TME) and secreted into the blood. Rising or elevated levels are indicative of cancer progression or recurrence and are useful for monitoring already diagnosed cases of breast cancer [87]. Historically, CEA is not recommended for pre-screening due to low specificity and is being replaced by more specific markers [88]. 

The first CEA-specific aptamer was produced in 2007 by Wang et al. [89]. The actual sequence of the DNA aptamer was not reported in the work. The group also felt that testing for affinity and K_d_ was unnecessary, and it is therefore not listed. The work focused on the specificity of the binding over the affinity to the ligand, finding that the aptamer could bind to both the native and purified ligand. 

The most common is CEAAp1 and has the sequence 5′-ATACCAGCTTATTCAATT-3 [90]. Parameters such as binding affinity, binding motif, and specificity of this DNA aptamer are severely lacking in the literature but use of this aptamer for sensing is common [90,91,92]. Using voltametric methods, sensors using aptamer CEAAp1 consistently get obtain high sensitivities in the Pico and femtogram range [91]. Work needs to be done to better understand whether the use of this aptamer is common due to standard or because it has actual benefit over other developed aptamers [93,94].

Two new DNA aptamers targeting the CEA antigen were recently reported by a group from Brazil [95]. These two aptamers, appropriately named Apta 3 and Apta 5, show high specificity and affinity for CEA-positive cell lines compared to CEA-negative cell lines. Apta 5 displays a K_d_ of 37.8 ± 5.8 nM, nearly half that of Apta 3, indicating better binding affinity. The specific binding motif of both aptamers is unknown at this time, but it is believed the binding site is similar between the aptamers. No work exists that tests these aptamers on complex matrices such as human serum, but this is unsurprising as the work is quite new.

### 3.5. HER2 

The human epidermal growth factor, commonly known as HER2, is a transmembrane glycoprotein found on the cell surface of tissue [87]. In tumors, the receptor is overexpressed in breast tissue. HER2 can also be found as circulating tumor cells (CTC) in the blood of approximately 26.9% of HER2+ patients [96]. Overexpression of HER2 occurs in 20–25% of all breast cancers (BC) [97,98]. In blood samples, a concentration of HER2 exceeding 15 ng/mL indicates HER2-positive breast cancer [99]. It is apparent why HER2 is considered a prognostic factor and indicates an increased metastatic potential, poor clinical outcomes, and a more aggressive BC subtype [99]. Identifying breast cancer as HER2-positive allows for targeted treatment and has been shown to improve survival rates compared to non-targeted treatment [100,101]. Multiple antibody assays are currently in use for HER2 detection, but many require specialized personnel to operate, leading to difficulties in quality assurance [102]. Many aptamers for HER2 exist [30,57]; for simplicity, they are herein divided by their specific target proteins.

### 3.6. HER2 Whole Protein

**Figure 2:**
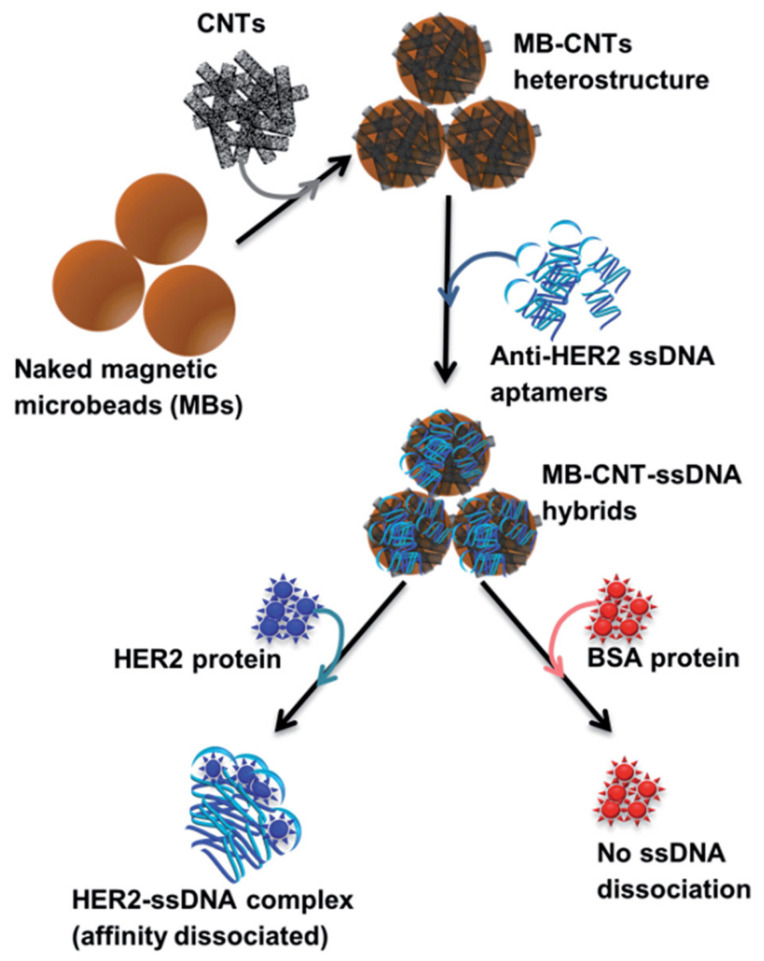
Schematic diagram showing sequential steps involved in in vitro method employed for HER2-specific targeted release/dissociation of H2 from MB–CNT–H2 cargos [104]. Note that the graphics of beads, ssDNA, or proteins shown in the scheme do not reflect their actual size or dimensions. (Reproduced with copyright permissions from 10.1039/C4AN01665C accessed on 31 May 2021).

The majority of HER2 aptamers target the entire protein and were developed using a whole-cell SELEX approach. Aptamers HeA2_1 and HeA2_3 were produced during the same study. Gijs and associates used the Dubbles concept, a process in which amplified DNA is heated and then rapidly cooled to produce ssDNA that is shorter in length, which was used to avoid the drawbacks of longer oligonucleotides. The cell line SKBR3 was used in the SELEX process as it is known to overexpress the HER2 protein. The K_d_ for HeA2_3 is a quarter of what it is for HeA2_1, indicating a greater binding affinity; however, both aptamers displayed rapid association and slow dissociation from the protein. Using three cell lines with varying expression of HER2 and a HER2 negative cells, the researchers determined that both aptamers are highly specific and block the binding of the natural antibody when in excess. The most interesting outcome of this study was that HeA2_3 was able to be internalized into HER2-overexpressing cells, showing great potential for targeted drug delivery [103]. The other DNA aptamer, H2, is reported to have a dissociation constant of 270 nM, which is significantly larger than both HeA2_1 and HeA2_3, indicating comparatively weaker binding. The authors were able to use the new aptamer to develop an assay for the detection of HER2 by wrapping the aptamer around carbon nanotubes measuring the affinity (Figure 2 [104]). Unlike the previously mentioned aptamers, the work on H2 had a greater focus on the aptamers’ potential for diagnosis, showing great promise in this area and demonstrating the ability to bind to both the native and free forms of HER2 [104]. 

The whole protein aptamers HeA2_1, HeA2_3, and H2 are DNA-based, unlike the RNA aptamer TSA14. TSA14 underwent the SELEX process and was derived from the TUBO cell line, a HER2-overexpressing derived from mouse mammary carcinoma. Unfortunately, the aptamer does not recognize TUBO cells exclusively, and the binding affinity is not as great as the RNA-based aptamers, indicating that TSA14 is an inferior aptamer to HeA2_3 [105]. One other RNA-based whole protein aptamer exists, entitled SE15-8. Aptamer SE15-8 exists both in a normal length and a minimal or “mini” version, with K_D_s of 10^−9^ M and 3.49 ± 1.3 × 10^−9^ M, respectively. Although the miniaturized version has a weaker binding affinity, its size means that it can more easily penetrate the cancer site [106]. 

### 3.7. HER2 Peptide 

Two aptamers exist that target the HER2 peptide specifically, both are DNA-based and less than 90 nucleotides in length. Both aptamers target a peptide with the sequence INCTHSCVDLDDKGCPAEQR, which is found in the juxtamembrane region of the extracellular domain of the peptide. Both aptamer HB5 [107] and HY6 [108] were found to bind specifically to HER2-positive cell lines and have a K_d_ for the whole protein of 316 and 172 nM, respectively. Aptamer HY6 underwent a modification of the phosphate backbone with phosphorothioate, making it a thioaptamer. This type of modification is known to enhance affinity and more importantly increase nuclease resistance. Due to shorter length, a lower K_d_ and subsequently great binding affinity, and increased nuclease resistance, aptamer HY6 is a better choice for diagnostic and treatment purposes.

### 3.8. HER2 Extracellular Domain

The remaining aptamers target the extracellular domain of HER2 and are DNA type. Heraptamer1 and Heraptamer2 were both produced using snap cooling, allowing them to obtain optimal conformation; a similar process was used for aptamers HeA2_1 and HeA2_3 [103,109]. Heraptamer1 and Heraptamer2 showed no cell toxicity towards HER2-positive tumor line SKOV3, indicating their use is more targeted towards imaging and diagnostics rather than therapeutics. With dissociation constants of 5.1 ± 5.3 and 23.7 ± 11.2 nM, respectively, Heraptamer1 and Heraptamer2 display a strong binding affinity and selectivity between HER2-positive and -negative cells. ECD_Apt1 is a similar aptamer produced by Sett et al. [110] that shows similar binding affinity with a K_d_ of 6.33 ± 0.86 nM. A benefit of this aptamer is that it has been tested in both solution and solid-phase immunoassays and was found to be sensitive in both. All three aptamers targeting HER2 ECD were published in 2017, and being relatively new, lack any studies used on clinical samples or complex matrices; the addition of such work would further advance the use of these aptamers.

### 3.9. VEGF 

Vascular endothelial growth factors (VEGF) are secreted glycoproteins and are key angiogenic factors, meaning that they stimulate the growth of new blood vessels [111]. When expressed in excess, VEGF is indicative of metastasis and tumor growth potential in many cancers, including breast [112]. Additionally, VEGF is immunosuppressive, hindering the body’s ability to recognize unregulated growth [113]. VEGF is overexpressed in many solid cancers as well as rheumatoid arthritis, meaning it is out of the question for use as a diagnostic marker. Studies have shown its potential as a predictive factor for overall survival and response to antiangiogenic treatment [114].

Aptamer Vap7 is one of two aptamers available that binds to multiple forms of VEGF. Vap7 binds to both VEGF-121 and VEGF-165, isoforms, which have similar prognostic values for breast cancer [115]. This aptamer targets the receptor binding domain of VEGF. Specificity for Vap7 was determined through a competitive binding assay with bovine serum albumin, thyroglobulin, and pyrroloquinoline quinone glucose dehydrogenase as competitors. This assay showed that Vap7 appears specific for VEGF. The structure of the aptamer was determined to be G-quadraplex, the first reported aptamer of that structure for VEGF, using CD spectroscopy. Binding affinity of the aptamer for VEGF_121_ and VEGF_165_ are similar at *K*_D_ = 1.0 nM and *K*_D_ = 20 nM, respectively [116]. After developing Vap7, the group went on to create a truncated mutant version entitled V7t1, which also bound to both isoforms of VEGF. V7t1 is a portion of the Vap7 aptamer and is significantly smaller at 29-mer and has a high affinity to both VEGF_121_ and VEGF_165_ (*K*_D_ = 1.1 nM and *K*_D_ = 1.4 nM, respectively). A heterodimer of the V7t1 aptamer was created using a previously developed aptamer (del5-1) that binds to the heparin binding domain of VEGF. With the heterodimers ability to bind to both the receptor binding and heparin binding domains, the binding affinity was significantly increased for VEGF_165_ with a *K*_D_ of 4.7 × 10^2^ pM.

RNV66 also binds multiple isoforms of VEGF and uses a locked nucleic acid (LNA) strategy to provide stabilization by resistance to nucleases and increased binding affinity [117]. In LNA, the ribose ring has a methylene linkage between the 2′-oxygen and 4′-carbon, thereby constraining the ring [118]. The benefit of LNA is that the resulting aptamer is better stabilized both in vivo and in vitro. This aptamer targets both VEGF_121_ and VEGF_165_ isoforms with high affinity and specificity and is based on the previously mentioned Vap7 aptamer. RNV66 was tested on both normal and cancerous epithelial breast cell lines and was found to be toxic to cancerous cells while having no significant effect on the normal breast cell line. The aptamer was also tested in vivo with mice models and was able to inhibit breast cancer progression [117]. This aptamer proves promising for therapeutic treatment of breast cancer and likely sensing as well due to its high specificity and affinity. 

The RNA aptamer pegaptanib sodium, commonly shortened to pegaptanib, binds only to the VEGF_165_ isoform, and selectively binds with the heparin-binding site [119]. At 27 nucleotides long, this aptamer binds in a calcium-dependent manner with a K_d_ of 49 pM [120]. Pegaptanib is historically noteworthy as it was the first aptamer to be approved for therapeutic use in humans, specifically to treat age-related macular degeneration [121]. The development of pegaptanib began in 1994 with the standard SELEX procedure for aptamer development, and some notable post-SELEX modifications make it the aptamer it is today. The aptamer underwent 2′-*O*-methylation to obtain increased nuclease resistance and stability, and the addition of 2′-fluoro-pyrimidine led to an even greater time in circulation [119,122]. To the authors’ knowledge, no studies exist where pegaptanib is used to sense or treat breast cancer, but some research hypothesizes its use to target invadopodium and subsequently block breast cancer metastasis [123].

**Figure 3:**
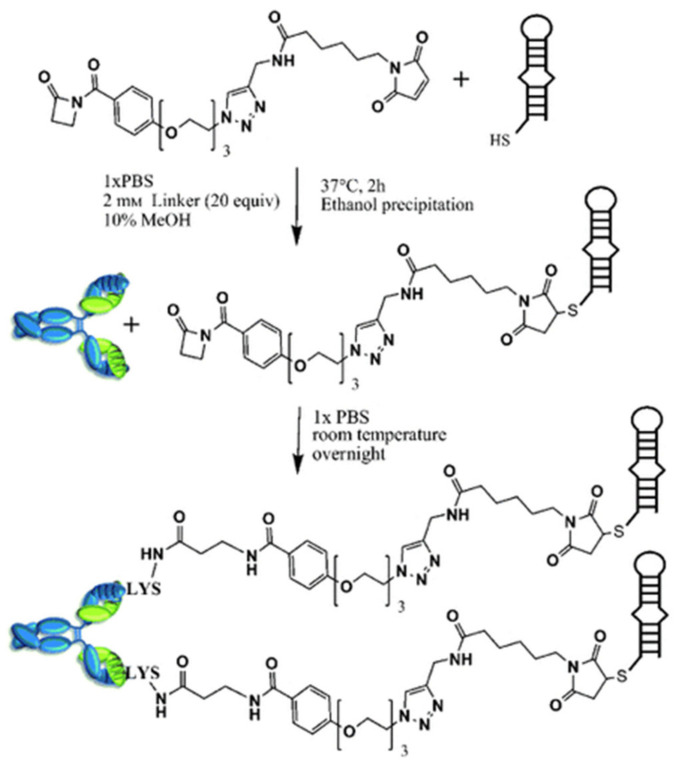
Irreversible programming of aldolase antibody 38C2 with aptamer ARC245 [124]. (Reproduced with copyright permissions from 10.1002/anie.201001736. accessed on 29 July 2021).

Another example of a 2′-*O*-methyl containing aptamer is ARC247. At 23 nucleotides in length, this aptamer exhibits a K_d_ of 2 nM and is extremely stable [120]. When conjugated to an antibody, the aptamer demonstrates increased affinity and time in circulation compared to the unconjugated version [124]. To achieve this, the researchers used a thiol-modified aptamer and β-lactam-based heterobifunctional linker to join the antibody and aptamer (Figure 3). The researchers also used a chemically programmed antibody, meaning they were able to retain some of the benefits of aptamers, such as small size and simplified manufacturing. This strategy is the first of its kind and is believed to be applicable to other aptamers. Work with this aptamer is relatively new, and no studies exist that use this aptamer on cancer cell lines.

Electrochemical aptasensors for the detection of VEGF is not a new concept, likely due to the well-studied nature of the biomarker itself. Advancements for the detection of VEGF have been made in the last five years [125,126,127] focusing on simple manufacturing and ease of use. Less work exists involving active testing of their sensors on clinical samples. The most promising work comes from Fu et al., wherein the authors were able to develop a label-free sensor that can be measured using both amperometry and cyclic voltammetry [128]. Unfortunately, like much of the work involving aptamers, the specific sequence is not listed [30]. The interaction of VEGF with the aptamer leads to the catalysis of the H_2_O_2_-mediated oxidation of 3,3′,5,5′-tetramethylbenzidine, resulting in increased current signal, but more interestingly, a blue color change in solution is observed. This color change indicates the possibility for a visual sensor similar to a pregnancy test. Continued work from the group is unfortunately lacking but the use for a commercialized sensor appears promising.

### 3.10. Progesterone Receptors

Part of the pathological evaluation of breast cancer is the evaluation of hormone receptor expression. Progesterone receptors, such as estrogen receptors, are one of the key hormone receptors that dictate treatment options. Unlike estrogen, the predictive/prognostic value of progesterone receptor expression is controversial [129]. The evaluation of progesterone receptor expression has been found to only be useful in a subset of patients already determined to be ER+ [130]. This gives an explanation as to why the authors were unable to find any progesterone aptamers for use in breast cancer. Exploration of progesterone receptor aptamers is impractical at this time as their predictive value is still highly debated and possibly overshadowed by that of estrogen receptors.

## 4. Conclusions

We have identified all known aptamers for specific biomarkers associated with breast cancer (Table 2) and compared the benefits and limitations of each. Our focus was on aptamers with specific molecular targets rather than cancer cell lines of tissues. We chose to do this as specific targets provide better diagnostic value and clinical application. Parameters of some aptamers are very limited, making them impractical for clinical use without further investigation.

For breast cancer, the number of developed aptamers is the most abundant for the HER2 target. This is likely since it is only overexpressed in breast cancer and indicates a very specific diagnosis. ERα demonstrates similar potential to HER2 due to its specificity towards BC; however, only one aptamer exists. Further improvement of the current aptamer or exploration of new ones is crucial for ensuring proper detection, which will lead to better targeted therapies. This is imperative as resistance to hormone therapies are easily developed.

More work should be done with ERα, as it is present in 75% of breast cancers and represents too large a portion of patients to be ignored. With an output time of only 10 min, the current available sensor shows great promise for diagnostic use. Further exploration should be done using complex matrices and serum samples to push this technology to the bedside.

If aptamers are to be used for the diagnosis and treatment of breast cancer, some factors should be focused on. It is crucial that greater cross-reactivity and clinical sample studies are conducted to ensure high specificity of these aptamers. Breast cancer treatments, such as chemotherapy, pose a very real harm to those who are in fact healthy and incorrectly diagnosed, and therefore the reliability of these aptamers must be held to the highest standard.

Although the FDA has approved multiple oligonucleotide therapies, at the time of writing, pegaptanib is the aptamer-specific therapy that is approved [131]. Challenges in pharmacokinetic properties have slowed therapeutic advancements in comparison to diagnostics, but modifications such as PEG are leading to better outcomes [132]. Work with aptamers for both diagnostic and clinical use is still promising, as a simple search of the National Institute of Health clinical database reveals that there are at least six currently active clinical trials registered.

As sensing platforms continue to develop, aptamers hold great potential for point-of-care diagnosis in the fight against breast cancer. Many aptasensors exist that exemplify proof-of-concept for use at the bedside and have been used on clinical samples [18], but to public knowledge, none are currently on market or being used as standard in clinical settings. Commercialization of aptasensors is proving to be a difficult feat, as antibody assays are well established, and educating investors and physicians on the benefits of aptamers can prove to be difficult. For hospitals, simple biosensor interfacing and the lack of specialized equipment should allow for simple point-of-care testing, thus reducing laboratory load and costs [133]. Although beneficial in the long term, this transition from a centralized lab to a decentralized point-of-care approach would require extra effort and expense.

Research on improving the viability of aptamers in the medical field is hindered by the lack of a regularly updated and functioning database for aptamers. Previous attempts have been made at these types of databases, but none have been successful and most no longer exist. The development of a database would accelerate aptamer work, allowing researchers to easily select the proper aptamer for their projects, make synthetic modifications, and run in silica calculations without additional hassle. 

Despite the work already achieved, further study and clinical trials are paramount in making the use of aptamers widespread and increasingly viable. With an increased focus on the development of new aptamers for targeting select cancer biomarkers, researchers should be able to leverage the high specificity and binding affinities of aptamers to develop potent drugs and sensing platforms. If completed, the use of aptamers for cancer diagnostics has the potential to provide a highly specific and sensitive platform that is simultaneously quick and user friendly. These aptamers display potential for not only the detection of cancers but can be further leveraged to target cancers for therapy. In either case, aptamers have the potential to help us make strides towards a world where treatment is quick to start and efficient in use.

## Figures and Tables

**Table 1:** The current available targets, their location, special characteristics, benefits for selection as a target, and challenges in use.

Target	Location	Characteristics	Benefits for Targeting	Challenges of Use
Alpha estrogen receptor	Mammary glands	Ligand-inducible transcription factor.	Found in 75% of breast cancers. Responsive to targeted hormone therapies.	Resistance to hormone therapy easily developed, proper identification critical to effective therapy.
Mucin 1	Expressed on circulating tumor cells	Transmembrane glycoprotein.	Indicator of cancer remission status. Large number of aptamers already exist. Overexpressed in 90% of breast cancers.	Overexpressed in multiple epithelial cancers, not exclusive to breast cancer.
Vascular endothelial growth factor	Endothelial cells	Secreted glycoproteins. Angiogenic factor—stimulates the growth of new blood vessels. Immunosuppressive.	Predictive factor for overall survival and response to antiangiogenic treatment.	Overexpressed in multiple solid cancers as well as rheumatoid arthritis. Not exclusive to breast cancer and cancers.
Periostin	Secreted into the tumor microenvironment	Multimodular protein.	Found overexpressed in up to 83% of invasive breast carcinoma patients [31].	Positive correlation between periostin levels and age; currently no normal range established.
Epithelial cell adhesion molecule	Trans- and intermembrane domains on epithelial and cancer cells [32]	Transmembrane glycoprotein. Responsible for cell adhesion, proliferation, and migration.	High expression on rapidly proliferating cells. Overexpression is seen in roughly 82% of metastatic breast cancers [33].	Higher expression seen in metastatic cancers. Poor viability for early cancer detection, thus shifting focus to late-stage cancer monitoring and drug targeting.
Nucleolin	Found primarily in the nucleolus	Phosphoprotein with RNA recognition motifs.	Predictive factor for multiple cancers, particularly potent prognostic factor for BC.	Expressed in multiple cancers, not specific to breast. High levels indicate poor outcomes for breast cancer but positive outcomes for other cancers.
Cancer antigen 15-3	Found in serum	Soluble product of the MUC-1 gene.	Can be used to distinguish bone metastasis from other types of metastases. Predictive factor for overall and disease-free survival. Indicator of metastatic potential in active treatment.	Current antibody-based sensors already exist with low LOD and short processing times.
Carcinoembryonic Antigen	Secreted into the tumor microenvironment	Secreted glycoproteins.	Elevated levels are indicative of cancer progression or recurrence.	Traditionally not recommended for screening due to low specificity.
Human epidermal growth factor 2	Transmembrane protein found overexpressed on breast cancer cells.	Transmembrane glycoprotein found in both tissue and circulating tumor cells	Overexpressed in 20–25% of breast cancers. Concentration exceeding 15 ng/mL in blood indicates HER2-positive breast cancer. HER2+ breast cancer is a more aggressive molecular subtype. Many aptamers already available.	Relatively low levels of expression.

**Table 2:** Summary of the known breast cancer targets, available aptamers for targeting biomarkers, the binding constants, and respective Gibbs free energy.

Target	Aptamer Name	Sequence 5′→3′	Association Constant (K_a_)	Dissociation Constant (K_D_)	ΔG (kcal/mol)
Alpha estrogen receptor	ERaptD4	ATACCAGCTTATTCAATTCGTTGCATTTAGGTGCATTACGGGGGTTATCCGCTCTCTCAGATAGTATGTGCAATCA	1.55 ± 0.29 × 10^8^ M^−1^	N/A	N/A
Mucin 1	5TR1	GAAGTGAAAATGACAGAACACAACA	0.20 × 10^7^ M^−1^s^−1^	47.3 nM	N/A
	S2.2	GCAGTTGATCCTTTGGATACCCTGG	0.401 × 10^7^ M^−1^s^−1^	0.135 nM	N/A
	MA3	AACCGCCCAAATCCCTAAGAGTCGGACTGCAACCTATGCTATCGTTGATGTCTGTCCAAGCAACACAGACACACTACACACGCACA	N/A	38.3 nM	N/A
Vascular endothelial growth factor	Vap7	ATACCAGTCTATTCAATTGCACTCTGTGGGGGTGGACGGGCCGGGTAGATAGTATGTGCAATCA	N/A	1.0 nM (121) and 20 nM (165)	N/A
	V7t1	TGTGGGGGTGGACGGGCCGGGTAGA	N/A	1.1 nM (121) and 1.4 nM (165)	N/A
	RNV66	TGTGGGGGTGGACGGGCCGGGTAGA	N/A	N/A	−9.34 28:28 10.34 (lowest listed)
	Pegaptanib	CGGAAUCAGUGAAUGCUUAUACAUCCG	N/A	49 pM	N/A
	ARC247	CGAUAUGCAGUUUGAGAAGUCGCGCAUUCG	N/A	2 nM	N/A
Periostin	PNDA-3	ACGAGYYGYCGCAYGYGCGGYYCAGYCYGGYCCYYCAGCACCGYACAACAA *Y, Benzyl-d(U)TP	N/A	1.07 nM	N/A
Epithelial cell adhesion molecule	SYL3	AGCGTCGAATACCACTACAGAGGTTGCGTCTGTCCCACGTTGTCATGGGGGGTTGGCCTGCTAATGGAGCTCGTGGTCAG	N/A	87 ± 17 nM (MDA-MB-231) and 185 ± 22 nM (Kato III)	N/A
	EpDT3	GCGACUGGUUACCCGGUCG	N/A	12.0 ± 6.5 nM	N/A
	EpApt-siEp	GCUGGCCCAUUGGUCAGC	N/A	N/A	N/A
Nucleolin	AS1411	TTTGGTGGTGGTGGTTGTGGTGGTGGTGG	N/A	N/A	N/A
Cancer antigen 15-3	Clone 2	GAAGTGAATATGACAGATCACAACT	N/A	45.47 ± 3.415 nM	N/A
	Clone 4	TACTGCATGCAGACCACATCAACTT	N/A	67.1 ± 3.289 nM	N/A
	Clone 5	CATACAATCAATCACCAGTGCGGTG	N/A	81.56 ± 4.198 nM	N/A
Carcinoembryonic antigen	Wang et al. (2007)	N/A	N/A	N/A	N/A
	CEAAp1	TTAACTTATTCGACCATA	N/A	N/A	N/A
	Apta 3	GGGGGGTGTATCGTTGACGAGTTGCGCGTGCGTCTCGTG	N/A	60.4 ± 5.7 nM	−18.69
	Apta 5	GGAGCTACGTTTAGCGAGTCCGACGCTCGGTGCCTCTTC	N/A	37.8 ± 5.8 nM	−13.06 kcal/mol
Human epidermal growth factor 2	HeA2_1	ATTAAGAACCATCACTCTTCCAAATGGATATACGACTGGG	N/A	28.9 nM	−7.82 kcal/mol
	HeA2_3	TCTAAAAGGATTCTTCCCAAGGGGATCCAATTCAAACAGC	N/A	6.2 nM	−9.27 kcal/mol
	H2	GGGCCGTCGAACACGAGCATGGTGCGTGGACCTAGGATGACCTGAGTACTGTCC	N/A	270 nM	−6.69 kcal/mol
	TSA14	GCUGGAGCAUUUAUGGAUGAACCUUGGACGGAA	N/A	133.9 ± 12.78 nM	−9.68
	SE15-8	AAAATACCAAATAAGGAAGCAAGGCAGAACGGGGCCTACTGTGTATATCATC	N/A	10 nM	N/A
	SE15-8 (mini)	AGCCGCGAGGGGAGGGAUAGGGUAGGGCGCGGCU	N/A	3.49 ± 1.3 × 10 nM	N/A
	HB5	AACCGCCCAAATCCCTAAGAGTCTGCACTTGTCATTTTGTATATGTATTTGGTTTTTGGCTCTCACAGACACACTACACACGCACA	N/A	18.9 nM (peptide) and 316 nM (ECD)	N/A
	HY6	TGCCCGTGTCCCGAGGAGGTGCCCTATTTTGCTTGATTATCTCTAAGGGATTTGGGCGG	N/A	183 nM (peptide) and 172 nM (ECD)	N/A
	Heraptamer1	ACACCACATCCGTCTTACTCCCCAATTACA	N/A	5.1 ± 5.3	N/A
	Heraptamer2	TCCACCTTTCCGTCTAACTCCCCACTTTAT	N/A	23.7 ± 11.2	N/A
	ECD_Apt1	CCGCAACCACGACCGAAAGACAACGCAATCTGACACGTGG	N/A	6.33 ± 0.86 nM	−3.24
